# What Is Rat-Bite Fever?

**Published:** 1917-08-11

**Authors:** 


### WHAT IS RAT-BITE FEVER?

The rat is an ugly customer and has a bad character, which he thoroughly deserves. He shows courage, it is true, and this makes him the more formidable. Apart from his physical and moral qualities, he cultivates other habits which set him at enmity with mankind. Some years ago it was discovered that he shelters the flea which is the agent engaged in the distribution of plague, and there are other charges against him. In particular, his bite, bad enough at the mildest, is able to give rise to a curious train of symptoms which are grouped under the term "rat-bite fever." Some cases have recently been reported in France, and one may perhaps speculate that subterranean and other disturbances, as well as an increased difficulty in obtaining food, have aggravated the beast's natural ferocity, and that thus rat bites and rat-bite fever may become more frequent. Japan seems to be the country where this special form of fever is best known, but a few instances have been recorded in this country; and there are special features of the disturbance which render its true nature readily overlooked unless the physician concerned happens to be posted in the matter. The original bite may have been a very small affair, and may have completely healed long before the constitutional evidences of rat-bite fever display themselves. Hence the event may be forgotten by the patient, who will naturally not be disposed to think that an illness occurring weeks or even months later can have any relation to a small and soundly healed wound inflicted long before. After this long incubation period there is a sudden rise of temperature and the usual symptoms of general febrile disturbance. In addition—and this may awake attention to the diagnosis—inflammatory signs appear in the neighbourhood of the local wound—pain, swelling, and enlargement of the neighbouring lymphatic glands. The patient is now very ill, and in a few instances he has become progressively worse and died. But in most cases both local and constitutional symptoms subside and convalescence appears to be established. Yet sooner or later the train of events repeats itself, it may be after an interval of a few days, or the postponement may extend over weeks or months. Back come the rigors, the high temperatures, and the rest, and this repetition may be presented on several occasions, so that the whole process is spread over weeks or months. These periodic febrile attacks are apt to be very puzzling, more especially as the general clinical examination is quite negative and no parasites can be found in the blood. There is no enlargement of the spleen, as in malaria, and the leucocyte count is usually definitely increased. How the symptoms are caused is uncertain. Some authorities suspect a protozoon, but no one has demonstrated it. The practical lesson seems to be that in recurring or periodic fever of unknown causation the possibility of rat-bite fever ought to be remembered. Is it possible that some of the fevers of the trenches are of this order?

